# Inhibiting CBX4 efficiently protects hepatocellular carcinoma cells against sorafenib resistance

**DOI:** 10.1038/s41416-020-01240-6

**Published:** 2021-01-21

**Authors:** Wei Zhao, Bo Ma, Zhihua Tian, Haibo Han, Jintian Tang, Bin Dong, Guo An, Baoshan Cao, Boqing Wang

**Affiliations:** 1 Key Laboratory of Carcinogenesis and Translational Research (Ministry of Education), Department of Cell Biology, Peking University Cancer Hospital and Institute, 100142 Beijing, P.R. China; 2 Key Laboratory of Carcinogenesis and Translational Research (Ministry of Education), Department of Lymphoma, Peking University Cancer Hospital and Institute, 100142 Beijing, P.R. China; 3 Key Laboratory of Carcinogenesis and Translational Research (Ministry of Education), Department of Central Laboratory, Peking University Cancer Hospital and Institute, 100142 Beijing, P.R. China; 4 Key Laboratory of Carcinogenesis and Translational Research (Ministry of Education), Department of Laboratory Center, Peking University Cancer Hospital and Institute, 100142 Beijing, P.R. China; 5 Department of Hepatopancreatobiliary Surgery, Affiliated Tumor Hospital of Xinjiang Medical University, 830011 Urumqi, Xinjiang P.R. China; 6 Key Laboratory of Carcinogenesis and Translational Research (Ministry of Education), Department of Laboratory Animal, Peking University Cancer Hospital and Institute, 100142 Beijing, P.R. China; 7 Department of Medical Oncology and Radiation Sickness, Peking University Third Hospital, 100191 Beijing, P.R. China

**Keywords:** Hepatocellular carcinoma, Nuclear receptors

## Abstract

**Background:**

This study aimed to investigate the possible role of inhibiting chromobox protein homologue 4 (CBX4) to deregulate of cancer stem cells (CSCs) and to evaluate the contribution of these molecules to sorafenib resistance in advanced hepatocellular carcinoma (HCC).

**Methods:**

HCC cell lines and a xenograft mouse model with resistance to sorafenib were employed to analyse the effects of miR424 on CSC characteristics. RNA expression was analysed by RT-PCR and next-generation sequencing in a cohort of HCC cancer patients and sorafenib-resistant (SR) cell lines, respectively, to validate the key microRNAs and targets in the network.

**Results:**

MicroRNA and mRNA profiles of SR cell lines identified miR424 and its direct target CBX4 as significantly associated with stem-cell-like properties, poor survival, and clinical characteristics. Functional experiments demonstrated that miR424 suppressed CBX4 and CBX4 induced nuclear translocation of YAP1 protein but was not associated with protein production. When YAP1 and CBX4 were modulated with CA3 and UNC3866, tumorigenicity and stem-like properties were extremely inhibited, thus indicating that these compounds exerted a strong anti-tumour effect in vivo against SR HCC cells.

**Conclusions:**

Our results revealed that blocking CBX4 expression is critical in response to sorafenib resistance with advanced HCC.

## Background

Hepatocellular carcinoma (HCC) is the third leading cause of cancer death worldwide (8.2% of all cancer-related deaths), with increasing incidence and high mortality.^1^ HCC is difficult to diagnose in its early stage and has poor survival because of its high frequency of recurrence, metastasis after hepatectomy, and resistance to common chemotherapy.^2^ Sorafenib, an oral multikinase inhibitor, is currently regarded as a first-line systemic treatment option in patients with advanced HCC due to its potential to provide a survival advantage of 2–3 months based on the results of two phase 3 clinical trials.^3,4^ Although the treatment has significant increased mean overall survival (OS), the high resistance rate has significantly limited the benefit of sorafenib therapy. Previous studies have reported that enrichment of cancer stem cells (CSCs) may contribute to sorafenib resistance after initial treatment years prior.^5,6^ However, the molecular mechanismes by which CSCs affect sorafenib efficacy in HCC are still unclear, and it remains to be elucidated whether CSCs play a role in the regulation of drug resistance in HCC. Therefore, exploring the development and evolution of targeted drug resistance is very important to improve the efficacy of HCC chemotherapy.

Chromobox homologue 4 (CBX4), also known as polycomb 2 (PC2) or NBP16, is located on chromosome 17q25.3 and encodes a polycomb repressive complex 1-associated protein (CBX4 protein) that is a member of the Polycomb group (PcG) of proteins.^7^ PcG proteins are transcriptional repressors that are mainly involved in regulating development, senescence, stemness, and cancer progression.^8^ The balance in PcG gene penetrance is crucial for proper stem cell homoeostasis and the prevention of CSC development. CBX4, a SUMO E3 ligase, is different from other members of the CBX family and prevents human epidermal stem cells from entering senescence and contributes to maintenance of their stemness.^7^ CBX4 increases the transcriptional activity of hypoxia-inducible factor 1α (HIF1α) and hypoxia-induced vascular endothelial growth factor (VEGF) expression and angiogenesis by promoting HIF1α SUMOylation.^9^ In addition, we previously reported that high CBX4 expression predicts poor OS in patients with HCC,^10^ which suggests CBX4 as an independent prognostic factor for HCC patients who received postoperative transarterial chemoembolisation treatment.^11^ Taken together, these results indicated that CBX4 may play a role in maintaining CSCs in HCC.

miRNAs have been identified as oncogenes or tumour-suppressor genes in regulating the progression of cancers. Recently, several miRNAs have been demonstrated to be associated with sorafenib resistance and to function as predictive biomarkers for the outcome of HCC patients receiving sorafenib treatment.^12–15^ For example, in HCC animal models, miR-221 upregulation is considered to be a molecular event associated with resistance to sorafenib. Thus pre-treated patients with low circulating miR-221 respond to sorafenib.^13^ Meanwhile, other studies have shown that some miRNAs are associated with the regulation of CSCs. For example, miR-613 inhibits liver CSC expansion by regulating the SOX9 pathway.^16^ Our previous study also showed that multiple miRNAs, including let-7c, miR-200b, miR424, and miR-222, are essential to maintaining stem cell-like properties by regulating PBX3.^17^

In this study, we demonstrated that miR424 could regulate CBX4 expression to maintain stem cell-like properties in sorafenib-resistant (SR) cells, and CBX4 overexpression was positively associated with the Hippo-YAP pathway in SR cells. Increasing CBX4 levels in HCC cells enhances HIF1α-dependent YAP1 nuclear translocation and induces sorafenib resistance, but blocking CBX4 and YAP1 with the CBX4 inhibitor UNC3866 and the YAP1 inhibitor CA3 significantly suppresses tumour cell growth and CSC properties, particularly in SR cells. In brief, our findings indicate that CBX4 is essential for tumorigenesis by initiating YAP1 function in the nucleus to maintain CSC capabilities and is a good therapeutic target for preventing and treating tumours as well as evaluating the prognosis in patients with SR HCC.

## Methods

### Cell lines, patient samples, and plasmids

The human HCC cell lines PLC and Huh7 were cultured in RPMI 1640 (Gibco, Grand Island, NY, USA) supplemented with 10% foetal bovine serum, 100 IU/mL penicillin, and 100 µg/mL streptomycin at 37 °C with 5% CO_2_ in a humidified atmosphere. The identities of the cell lines were verified by DNA fingerprinting, which was performed by short tandem repeat DNA profiling. One hundred and six paired tumour and adjacent nontumour samples were obtained from HCC patients who underwent hepatectomy with an agreement at the Department of Hepatology Surgery from the ATH of XJMU (Affiliated Tumor Hospital of Xinjiang Medical University). The acquisition and use of these tissues were permitted based on the acquisition of informed consent according to the protocol approved by the Ethics Committee (no. G-201419). Validated pCDNA3.1-CBX4 and PLKO.1-shCBX4 (5’-CCGGCGTGATCGTGATGAGCAAATACTCGAGTATTTGCTCATCACGATCACGTTTTTG-3’) plasmids were kindly gifted by Professor Tiebang Kang from Sun Yat-sen University Cancer Center, Guangzhou, China and cloned into the lentivirus shuttle vector plenti6 (Invitrogen). Various lentiviruses were packaged in 293T cells by ViraPower Packaging Mix (Invitrogen) according to the manufacturer’s instructions as described in our previous study.^18^

### Establishment of SR cell lines

To establish SR subclones, Huh7 and PLC parental cells were cultured with various concentrations of sorafenib for 6 weeks, and surviving cells were passaged 4 times and constantly incubated with 10 µmol/L sorafenib. The establishment of these resistant subclones was conducted prior to performing the experiments.

### Cell growth inhibition assay

SR cells and their corresponding parental cells were cultured in 96-well plates with 0.0001–100 µmol/L sorafenib for 72 h. Cell viability was then assessed using the CellTiter 96 aqueous nonradioactive cell proliferation assay (3-(4,5-dimethylthiazol-2-yl)-5-(3-carboxymethoxyphenyl)-2-(4-sulfophenyl)-2H-tetrazolium) according to the manufacturer’s instructions (Promega). The results are presented as the percentage of control and were repeated at least three times.

### The luciferase reporter assay

The 3’-UTRs of CBX4 carrying the putative miR424-binding sites or mutant-binding sites were amplified by polymerase chain reaction (PCR) and inserted immediately downstream of the firefly luciferase cDNA in the pGL3-control vector (Promega, Madison, WI, USA) to construct pGL3-CBX4 wild type (WT) and pGL3-CBX4 MUT. Briefly, 10^5^ cells per well were seeded in 24-well plates, and 300 ng of pGL3 constructs plus 26 ng of pRL-TK plasmid that expressed Renilla luciferase were co-transfected with 60 pmol of miR-424 mimics or miR-424 mimics control (GenePharma) using Lipofectamine 2000 (Invitrogen). After transfection for 48 h, the luciferase activity was measured using a Dual-Luciferase Assay Kit (Promega). The data for each sample were normalised to Renilla luciferase activity, and three independent experiments were performed.

### RNA extraction and quantitative real-time PCR (qRT-PCR)

Total RNA was extracted from cultured cells using QIAzol (Qiagen, Hilden, Germany) for both miRNA and mRNA analyses. For mature miRNA quantification, 1 μg of total RNA was subjected to the addition of poly(A) tails by poly(A) polymerase (NEB, Beverly, MA, USA), followed by reverse transcription with an oligo (dT) adaptor primer. For mRNA detection, cDNAs were synthesised from 4 μg of total RNA using oligo (dT15) primers. For the analysis of mature and CBX4, qRT-PCR was performed using the above cDNA with SYBR Green PCR Master Mix (Applied Biosystems, Foster City, CA) and the appropriate primers (nucleotide sequences are provided in Table S1) on an ABI Prism 7500 Fast (Applied Biosystems) according to the manufacturer’s instructions as previously reported. Data are presented as relative quantification to U6 or glyceraldehyde 3-phosphate dehydrogenase based on calculations of 2^−ΔCt^ where ΔCt = Ct (Target) − Ct (Reference). Fold change was calculated by the 2^−ΔCt^ method.

### Spheroid-formation assay

To assay sphere-formation efficiency, 100 cells per well in a single-cell suspension were plated in ultra-low attachment 96-well plates (Corning Incorporated Life Science, Acton, MA, USA) and cultured in 100 μL of 1:1 mix of 2% methylcellulose (Sigma) and Dulbecco’s modified Eagle’s medium/F12 supplemented with 50 ng/mL epidermal growth factor (EGF), 50 ng/mL basic fibroblast growth factor (FGF), 10 ng/mL hepatocyte growth factor (HGF), and B27 (1:50) (Invitrogen) according to the protocol as previously reported.^6^ After the plates were incubated at 37 °C under a 5% CO_2_ atmosphere for 2–3 weeks, the spheres >100 μm in diameter were counted under a stereomicroscope (Olympus, Tokyo, Japan).

### Western blot analysis

Total protein was extracted from cultured cells using RIPA (ShineGene Molecular Biotech, Inc., Shanghai, China) according to the manufacturer’s instructions. Sodium dodecyl sulfate–polyacrylamide gel electrophoresis and western blotting were performed using standard protocols. The primary antibodies and the secondary horseradish peroxidase (HRP)-conjugated goat anti-mouse or anti-rabbit antibodies used as well as the corresponding dilutions are listed in Table S2. Signals were detected using the Immobilon^TM^ Western Chemiluminescent HRP substrate (Millipore).

### Indirect immunofluorescence staining

Huh7 and genetic cells as well as primary cancer tissues were subjected to indirect immunofluorescence staining with YAP1 (1:100), HIF1α (1:100), and CBX4 (1:100) primary antibodies followed by labelling with Alexa-488 (for CBX4 or HIF1α and YAP1) and Rhodamine (for YAP1) as described elsewhere.^19^ Nuclei were stained with 4,6-diamidino-2-phenylindole dihydrochloride (Polysciences, Warrington, PA, USA) at 0.5 μg/mL. All specimens were mounted in 90% glycerol/phosphate-buffered saline with 2.5% 1,4-diazabicyclo(2,2,2)octane and assayed by confocal microscopy (SP5, Leica, Wetzlar, Germany).

### In vivo xenograft mouse model

To establish a tumour xenograft mouse model in order to assess tumorigenicity, a dilution series of Huh7, Huh7-SR, PLC, and PLC-SR cells were mixed with an equal volume Matrigel (10 mg/mL, BD, Biosciences, Bedford, MA, USA), and 100 μL of the suspensions was subcutaneously (s.c.) injected into the backs of 4–6-week-old female BALB/c nude mice, which were kept housed in a specific pathogen-free class experimental animal room with a clean air conditioning system (Vital River Laboratory Animals, Beijing, China). Approximately 10 weeks later, the frequency of tumour formation was calculated based on extreme limiting dilution analysis using the webtool at http://bioinf.wehi.edu.au/software/elda/.^20^ Tumour tissues were sectioned and frozen at −80 °C. To measure tumour growth, all SR and parental cells (10^6^ cells in each mouse, random 5 mice for each group) were s.c. injected into mice for 3 weeks; when the value of tumour volume >50 mm^3^, 10 mg/kg/day sorafenib was administered via mouth for 9 days. To detect the effects of CBX4 and YAP1 inhibitors, mice bearing Huh7-SR xenografts underwent intraperitoneal (i.p.) injection of CA3 at 1 mg/kg,^21^ UNC3866 at 10 mg/kg,^22^ or both drugs every 2 days for a total of 2 weeks. All tumour growth was monitored three times a week. Mice were sacrificed by CO_2_ rapidly without suffering, and the tumours were dissected at the end point. All animal experiments were approved by PUCH and conformed to the regulatory standards of PUCH on Laboratory Animals Care and Use in accordance with the National Institutes of Health Guide (Guide for the Care and Use of Laboratory Animals, 2011).

### Statistical analysis

All data in the figures are presented as the mean ± SD, in which data are presented as indicated. The significance of differences between two groups was determined using a two-sided Student’s *t* test unless otherwise specified. In case of multiple tests, one-way analysis of variance followed by Bonferroni–Holm procedure was applied. Survival curves for patients were plotted using the Kaplan–Meier method, with the Mantel–Cox test for statistical significance. All data were analysed with the SPSS 20.0 statistical software (IBM, Chicago, IL, USA). *p* < 0.05 was considered statistically significant.

## Results

### SR cells highlight the CSC properties of HCC

**Fig. 1:**
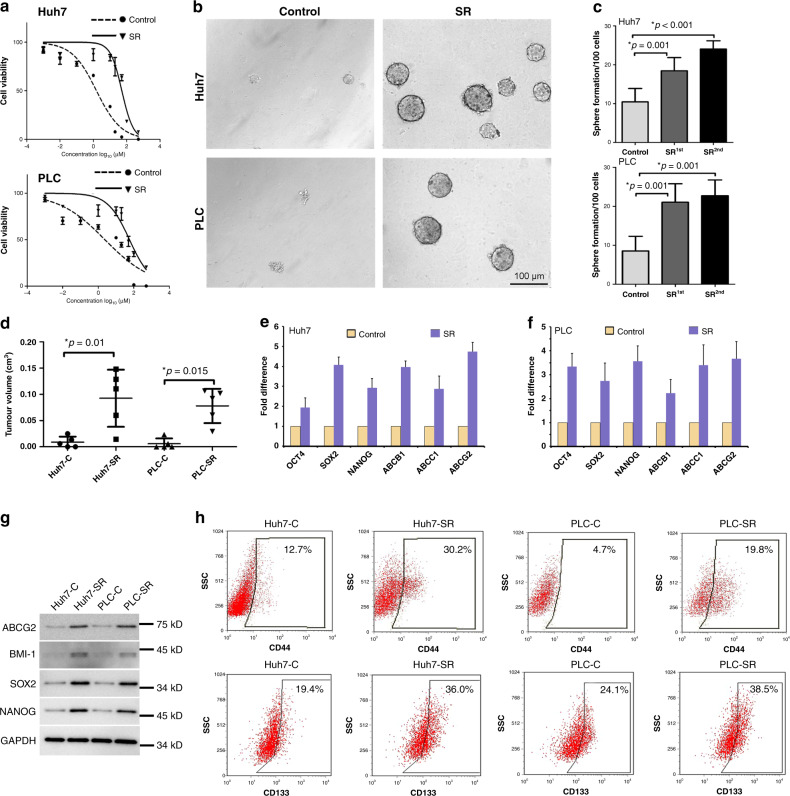
Sorafenib resistance induces cancer stem cell properties. **a** Huh7-SR and PLC-SR cells were established after 6 weeks of constant treatment with 10 µmol/L sorafenib. MTS was performed to calculate cell proliferation based on sorafenib treatment. **b** Representative phases show the spheroids formed from SR cells and parent cells. **c** The ability of the spheres formed by SR cells to form secondary spheroids was also shown. Spheroids (Φ > 100 μm) were counted under a stereomicroscope. **d** Serial transplantation was performed to analyse tumour growth of xenografted tumours derived from SR cells and parent cells, and the results are shown in Table S3. **e**, **f** qRT-PCR analysis of the expression of stem cell markers and drug resistance-related genes in SR and parental cells. Data are presented as the fold difference over parent cells for each gene, which was defined as **e** for Huh7 and **f** for PLC. **g** Stem-associated factors were tested by western blot. **h** CD44 and CD133 were validated between the parent and SR cells using FACS.

To assess whether resistance occurs in HCC therapeutically treated with sorafenib, we established the SR cell lines Huh7-SR and PLC-SR. As shown in Fig. 1a, compared with the parental controls, Huh7-SR (IC50, from 1.54 ± 0.18 to 50.9 ± 1.7 μmol/L) and PLC-SR (IC50, from 2.561.54 ± 0.37 to 56.9 ± 1.8 μmol/L) had significantly enhanced proliferation in the presence of sorafenib in a dose-dependent manner. Using three-dimensional culturing, sorafenib resistance facilitated sphere formation in both Huh7-SR and PLC-SR cells (Fig. 1b), and secondary tumour formation was also remarkably increased (Fig. 1c). Furthermore, the self-renewal tumour-formation capability in vivo was performed by detecting CSC properties in cells with sorafenib resistance (Fig. 1d). The frequency of successful transplantation of the diluted series of cells was affected by sorafenib resistance (Table S3). The tumour-forming ability of Huh7-SR and PLC-SR cells was dramatically increased both in vitro and in vivo. In addition, the expression of a panel of stem cell-like genes such as *OCT4*, *NANOG*, and *SOX2* and the ATP-banding transfer genes *ABCG2*, *ABCC1*, and *ABCB1* was highly amplified in Huh7-SR and PLC-SR cells compared with their parental controls as measured by qRT-PCR (Fig. 1e, f). Furthermore, stem-like proteins were upregulated both in Huh7-SR and PLC-SR cells by western blot (Fig. 1g). Moreover, stem cell markers, CD44 and CD133, were performed to detect the expression on the surface of difference cells between the control and SR groups (Fig. 1h). It shows that sorafenib resistance cells have occupied more CSC cells. In addition, migration of SR cells was obviously increased compared with parental cells (Fig. S1). These data indicate that sorafenib resistance induces HCC stem cell-like characteristics and enhances HCC tumorigenesis and migration.

### CBX4 is related to sorafenib resistance

**Fig. 2:**
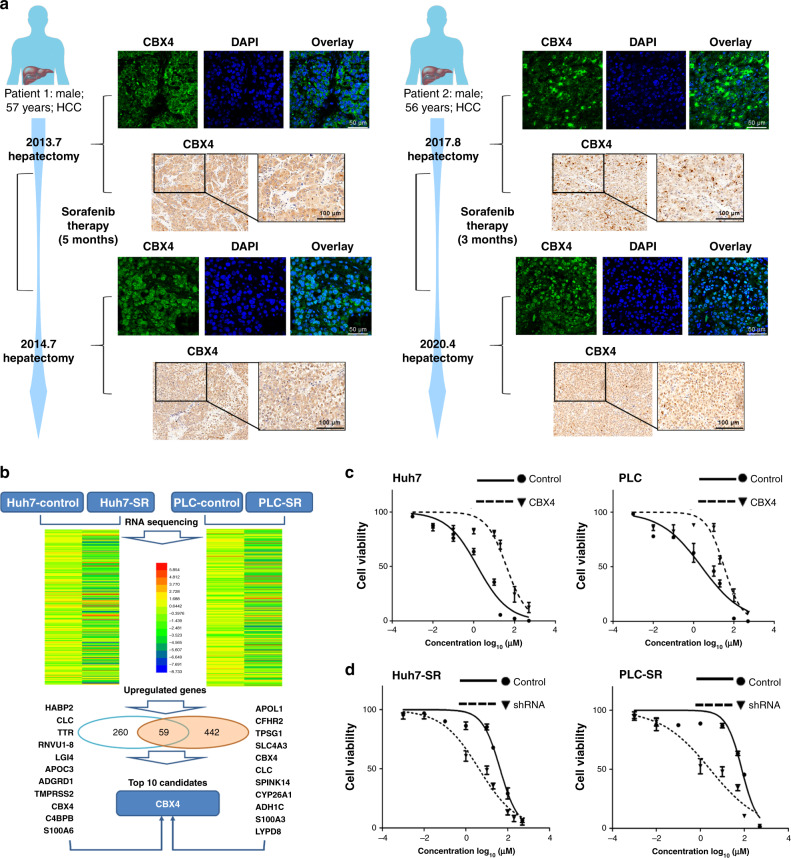
CBX4 is a candidate gene that contributes to sorafenib resistance. **a** Immunofluorescence and immunohistochemistry staining were performed between primary and recurrent tumour tissues from two patients who had failed in sorafenib treatment. **b** Representative chart showing CBX4 expression based on RNA sequencing of SR cells and parent cells. CBX4 is presented as one of the top 10 selected genes from 59 total upregulated genes in both Huh7 and PLC-SR cells. **c** The cell proliferation assay was performed by MTS in CBX4-modified cells and their control cells. **d** Conversely, SR cells with CBX4 knockdown were more sensitive to different doses of sorafenib. The IC50 values are shown in Table S4.

A previous study revealed that CBX4 served as a novel prognostic predictor and contributed to the strategy of HCC therapy.^10^ However, we do not fully know the relationship between CBX4 and sorafenib resistance. In this study, we found two recurrent HCC patients who had undergone the twice hepatectomies and constantly treated with sorafenib about 3–5 months between two surgical operations (Fig. 2a). Sorafenib was involved in the translocation of CBX4 from the cytoplasm at primary tumours to the nucleus at recurrent tumours. We then tested RNA sequencing between Huh7-SR and PLC-SR cells and their respective parental cells to identify epigenetic changes related to sorafenib resistance. A heat map was generated, and top genes that exhibited overall upregulation in SR cells were identified. Interestingly, CBX4 was upregulated 5.28-fold in Huh7-SR cells (sixth of the 442 upregulated genes, logFC >2, *p* < 0.05) and 2.7-fold in PLC-SR cells (ninth of the 260 upregulated genes, logFC >2, *p* < 0.05) (Fig. 2b). Hence, we decided to transduce CBX4 into parental cells and shCBX4 into SR cells to find a correlation between CBX4 expression and sorafenib resistance. With full-length CBX4 cloned into a lentivirus system for obtaining cells with stable overexpression of CBX4, we found that CBX4 overexpression drives more proliferation in the presence of 0.001–500 µm/mol sorafenib (Fig. 2c). In contrast, knockdown of CBX4 was used to test the sorafenib-dependent influence on proliferation (Fig. 2d). This evidence implicated that a higher sorafenib IC50 value was exhibited in CBX4-overexpressing cell and SR cell, as described in Table S4.

### miR424 mediates the suppressive effect on CBX4 gene expression

**Fig. 3:**
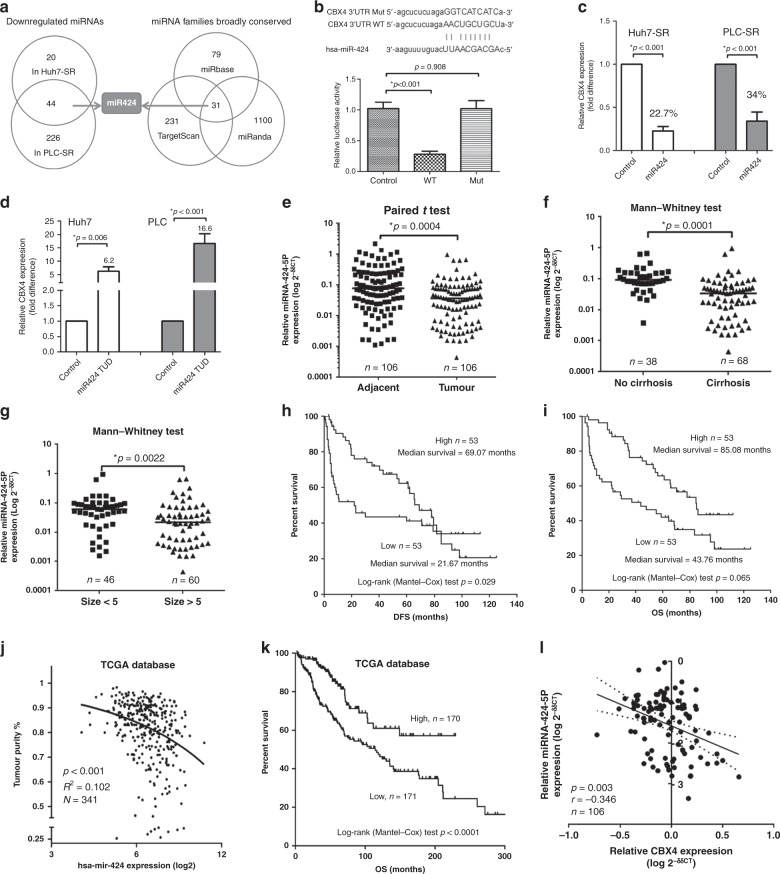
CBX4 is a direct target of miR424 and inversely correlated in HCC tissues. **a** The diagram illustrates how miR424 was identified from the miRNA sequencing data and predicted by Venn screening from miRNA database websites. **b** Sequence alignment of the human miR424 seed sequence with the 3’-UTR of CBX4. The mutated sequence in the matched binding sites for the gene that was used to create the firefly luciferase reporter constructs is shown at the bottom of the gene set. A luciferase reporter assay demonstrated that miR424 inhibited the transcription of the wild-type but not the mutant 3’-UTRs of CBX4. **c** The expression of endogenous CBX4 was inhibited in miR424-overexpressed Huh7-SR and PLC-SR cells. **d** In contrast, CBX4 levels were increased in Huh7 and PLC cells with miR424-TUD. All data were compared with the respective controls, and the mRNA level was detected by qRT-PCR. CBX4 mRNA expression was normalised to that of GAPDH mRNA; and three independent experiments were conducted. **e** Relative miR424 expression in HCC tissues and matched adjacent normal tissues as assessed by qRT-PCR. **f**, **g** Relative expression data of miR424 in HCC cases were further analysed. The negative relationship between miR424 expression and liver cirrhosis (**f**) and size (**g**). **h**, **i** Kaplan–Meier curves of disease-free survival (DFS) (**h**) and overall survival (OS) (**i**). Survival of the high and low miR424 expression groups assessed using log-rank (Mantel–Cox) test in HCC, which were divided according to a cut-off of 2.5, the median value of CBX4 mRNA expression relative to GAPDH mRNA. **j** From the TCGA database, tumour purity was highly negatively correlated with miR424 expression. **k** Linear regression and correlation between the miR424 and CBX4 mRNA levels in 341 HCC tissues from the TCGA database. **l** The negative linear regression and correlation analysis for the relation of the mRNA levels of CBX4 and those of miR424 in 106 HCC patients by qRT-PCR.

To gain insight into the reaction of CBX4-induced sorafenib resistance, miRNA sequencing analysis between SR and parental cells was performed to confirm which miRNAs were upregulated or downregulated in response to sorafenib resistance. In addition, 226 miRNAs and 203 miRNAs were significantly downregulated in PLC-SR and Huh7-SR, respectively. Forty-four of co-downregulated miRNAs are listed in Table S5. Based on 31 of predicted miRNAs conserved with CBX4 corresponding from 3 miRNA website analyses (miRbase, TargetScan, and miRanda, Table S5), miR424 is a candidate upstream regulator of CBX4 that affects the response to sorafenib resistance (Fig. 3a). To understand whether CBX4 is a direct target of miR424, a luciferase reporter assay was performed with vectors containing the 3’-untranslated region (3’-UTR) of CBX4 with the putative binding sites of miR424 (Fig. 3b, Up). Compared to co-transfection of the 3’-UTR with miR-WT or miR-Mut, transfection of the 3’-UTR with miR424 led to a significant decrease in luciferase activity in 293FT cells (Fig. 3b, Down). Furthermore, qRT-PCR analysis confirmed that CBX4 expression decreased by approximately 77.3 and 65.9% in miR424-overexpressed Huh7 and PLC cells, respectively, compared with corresponding control cells (Fig. 3c). This inverse relationship between miR424 and CBX4 mRNA is causal because ectopic miR424 expression reduced CBX4 mRNA levels, whereas treatment with an miR424-TUD blocked the upregulation of CBX4 mRNA (Fig. 3d). To further assess the function of miR424 in cells regarding the rescue of CBX4-induced sorafenib resistance, we measured the IC50 value of sorafenib in CBX4-overexpressing Huh7 and PLC cells and CBX4 knockdown Huh7-SR and PLC-SR cells (Table S4), even after additionally infected with miR424 or miR424 TUD. As shown in Table S6, miR424 definitely increased the resistance of HCC cells to sorafenib through CBX4 interaction. These data demonstrate that ectopic expression of miR424 downregulates the endogenous expression of CBX4 and then enhances sorafenib drug sensitivity in HCC.

### Clinicopathologic characteristics and bioinformatics analysis of miR424

Because we demonstrated that miR424 remarkably contributes to stem cell-like properties in HCC,^17^ the relative expression data of miR424 in a total of 106 cases were further analysed. Relative to the expression of U6, the level of miR424 was significantly downregulated in HCC tissues compared with matched adjacent normal tissues (Fig. 3e). To determine the correlation between the clinicopathological characteristics and the levels of miR424 in HCC, the data of all the included patients are summarised (Table S7). No significant correlation was observed between miR424 expression levels and gender, age, or venous invasion. However, miR424 expression in the HCC tissues with local tumour cirrhosis was significantly lower than that in tissues without tumour cirrhosis (Fig. 3f). Additionally, miR424 expression was markedly decreased in tumours >5 cm (Fig. 3g). Next, we analysed Kaplan–Meier curves and discovered that miR424 expression is associated with longer mean disease-free survival (DFS) and OS in HCC patients (Fig. 3h, i). Using data from The Cancer Genome Atlas (TCGA) database and running it through the LinkedOmics website, we validated the high expression of miR424, which was negatively and significantly associated with tumour purity (Fig. 3j), as well as its correlation with good OS outcomes (Fig. 3k). Because CBX4 contributed to poor clinical outcome in HCC that we had been reported previously,^10^ the correlation between the mRNA level of CBX4 and miR424 (*r* = −0.346, *p* = 0.003) was shown with negative relationship (Fig. 3l). These results suggest that low miR424 expression might be a candidate indicator of poor prognosis in HCC patients.

### miR424 suppresses proliferation by inhibiting CBX4 associated SR cells in a xenograft nude mouse model

**Fig. 4:**
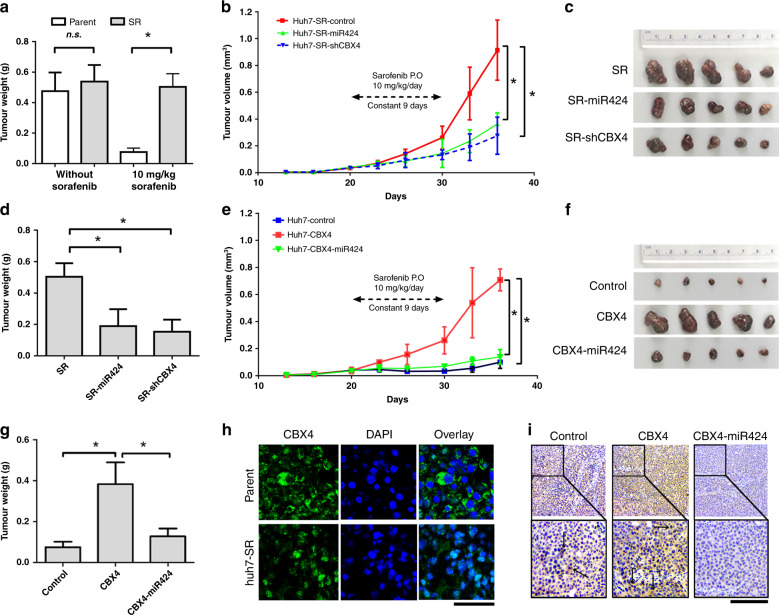
miR424-CBX4 affects tumour growth in a sorafenib-resistant cell-derived xenograft nude mouse model. **a** Tumour weight was measured and showed that tumour growth with or without sorafenib treatment. **b** Growth curves of subcutaneous xenografts derived from various genetically altered Huh7 cells (*n* = 5 per group). **c** Image of five xenograft tumours per group of Huh7-SR control and genetically infected cells with miR424 and shCBX4 after sorafenib treatment. **d** Tumour weight was measured and showed that Huh7-SR cells with sorafenib treatment and in genetically infected cells with miR424 and shCBX4. **e**, **f** Growth curves (**e**) and tumour images (**f**) of subcutaneous xenografts derived from Huh7-CBX4 overexpression cell and Huh7-CBX4-miR424 overexpression cell and control cell. **g** Tumour weight of CBX4-induced Huh7 growth and rescued by the inhibition of miR424. **h** Representative images of immunofluorescence (IF) staining of CBX4 in frozen tumour tissues treated with sorafenib showed the different localisations of CBX4 in Huh7-SR tumours and parental cell tumours, bar = 100 µm. **i** Expression of CBX4 in nucleus (black arrow) and cytoplasm from paraffin-embedded tumour tissues is shown and was rescued by miR424 expression based on immunohistochemistry, bar = 200 µm. * means *p* < 0.05 by one-way ANOVA test, except for the panel "a" by Student’s t-test.

To evaluate whether CBX4 and its upstream regulator miR424 affect sorafenib resistance in HCC in vivo, we examined tumour growth with variant genetically altered cells by constantly treating mice with sorafenib (10 mg/kg/day) via oral administration for 9 days. As shown in Fig. 4a, the tumour growth of Huh7-SR cells was dramatically increased compared with that of the Huh7 parental cells with sorafenib treatment; however, without sorafenib, the tumour formation in mice of Huh7-SR and parental cells was not significant. This tumour proliferation was suppressed by approximately 62 and 70% upon treatment with miR424 and CBX4 short hairpin RNA (shRNA), respectively, although the percentage of tumour inhibition by treatment with both miR424 and shRNA was varied (Fig. 4b–d). Furthermore, with sorafenib treatment, CBX4 overexpression in Huh7 rapidly drives tumour growth; however, this characteristic was specifically inhibited by as much as 85% when Huh7 CBX4-overexpressing cells were treated with miR424 (Fig. 4e–g). Immunofluorescence showed that CBX4 is weakly expressed in the nuclei of Huh7 cells in response to sorafenib treatment but is still maintained in the nucleus of Huh7-SR cells even in the presence of sorafenib (Fig. 4h). Furthermore, haematoxylin and eosin and immunohistochemistry were performed to validate CBX4 expression in paraffin-embedded tumour tissues from mice (Fig. S2 and Fig. 4i). The immunostaining for CBX4 in the cytoplasm and nucleus of the CBX4 overexpression group was much stronger than that in control Huh7 cells after sorafenib treatment and was highly downregulated in the CBX4-miR424 overexpression group. These results demonstrated that miR424 plays a critical role in inhibiting CBX4 expression during the progression of sorafenib resistance in HCC.

### Hippo-YAP associates with CBX4 by clustering the signal pathway

**Fig. 5:**
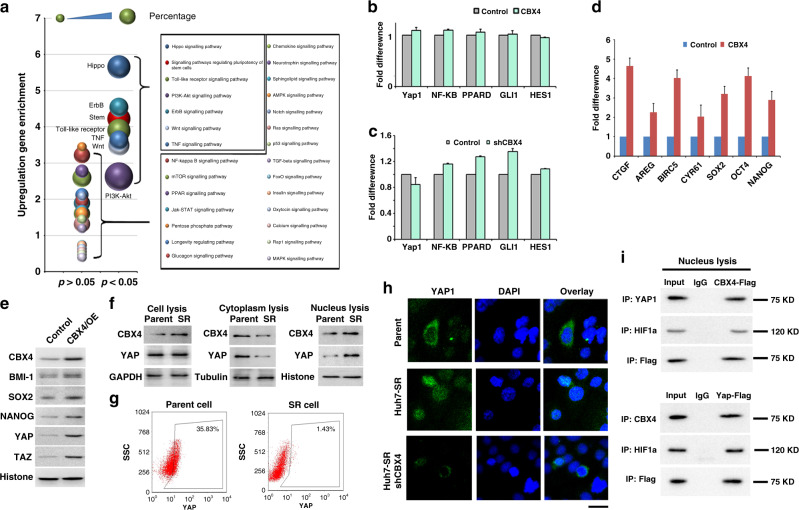
The Hippo-YAP pathway is an important pathway downstream of CBX4. **a** The KEGG analysis chart shows the enriched pathways in response to unregulated genes in Huh7-CBX4 cells. The significant group of pathways is separated from a total of 28 signalling pathways; this group includes the Hippo, Toll-like receptor, ERBB, WNT, TNF, stem-like, and PI3K-AKT pathways. **b**, **c** Histogram shows gene amplification by qRT-PCR in CBX4 genomic cells (**b**) and CBX4 knockdown cells (**c**). **d** The genes downstream of the YAP pathway were amplified by qRT-PCR. **e** Stem-associated proteins were tested in nucleus by western blot. **f** The protein level of YAP1 and CBX4 in whole cell, cytoplasm, and nucleus between SR and parental cell. **g** Qualification of the YAP1 expression in cytoplasm between SR cell and control using flow cytometry. **h** Representative images show YAP1 expression and localisation in parent cells, SR cells, and SR-shCBX4 cells by IF, bar = 100 µm. **i** Immunoprecipitation and immunoblotting analyses were performed with the indicated CBX4–YAP1–HIF1α interaction in the nucleus.

**Fig. 6:**
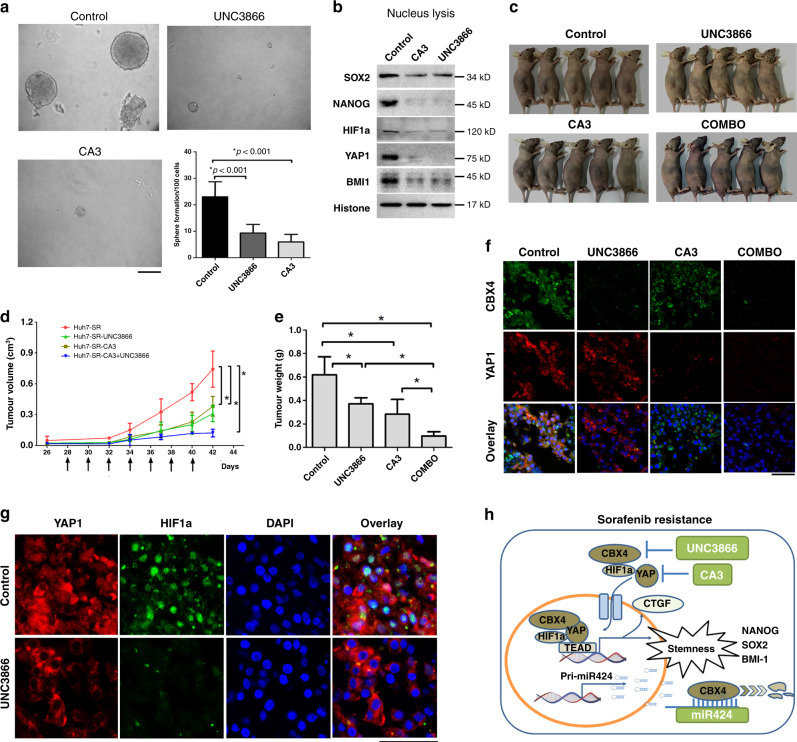
The combination of CA3 and UNC3866 suppresses tumorigenicity in vitro and in vivo. **a** Representative phases show the spheroids formed after treatment with CA3 and UNC3866 in Huh7-SR cells. The ability of the cells to form spheres was also shown. Spheroids (Φ > 100 μm) were counted under a stereomicroscope, bar = 100 µm. **b** The protein level in the cell nucleus was validated using western blot analysis, and histone H3 was used as an internal control. **c** After tumours were visible, mice with subcutaneously transplanted Huh7-SR cells were i.p. injected with PBS + DMSO, CA3, UNC3866, or CA3 plus UNC3866 (COMBO) at the indicated doses every other day for a total of 2 weeks. **d** Growth curves of Huh7-SR engraftment of each treatment group, per mouse as indicated by arrows. **e** The histogram shows the weight of the dissected tumour at experiment termination. *p < 0.05 by Student’s *t* test. **f** CBX4 and YAP1 expression and localisation in frozen tumours from mice were identified by double IF, bar = 100 µm. YAP1 is red; CBX4 is green; and the nucleus is blue (DAPI staining). **g** Immunofluorescences staining analysed YAP1 and HIF1α expression at nucleus and cytoplasm in Huh7-SR between the control group (PBS treatment) and UNC3866 group (CBX4 inhibitor treatment). Bar = 100 µm. **h** A diagram of the regulatory pathway of CBX4 and its constituents that determines the mechanism of sorafenib-resistant HCC. * means *p* < 0.05 by one-way ANOVA test.

To further understand the signalling pathway involved in the response to sorafenib resistance in HCC, RNA sequencing data between Huh7-CBX4 cells and control cells were analysed by Kyoto Encyclopedia of Genes and Genomes (KEGG). Three conditions (percentage of whole genes, number index of signalling genes and *P* value of each pathway) were considered to find the signal pathway candidates that influence sorafenib resistance. Several cell signalling pathways were enriched in CBX4-overexpressing cells. Interestingly, the Hippo pathway, Toll-like receptor pathway, ERBB pathway, etc. were clustered into the top five (Fig. 5a). Because the Hippo pathway was the most enriched in CBX4 cells, the mRNA level of Yap1, as well as of several other signalling molecules, was detected by qRT-PCR in CBX4-overexpressing cells. Unfortunately, compared with *NF-κB*, *PPARD*, *GLI1*, and *HES1*, *YAP1* was not obviously upregulated by CBX4 (Fig. 5b). Similarly, YAP1 was not sufficiently suppressed in CBX4 knockdown cells (Fig. 5c). To clarify that the Hippo-YAP pathway is a reasonable target of CBX4, the downstream genes of YAP1,^23^ *CTGF*, *AREG*, *BIRC5*, *CYR61*, *SOX2*, *OCT4*, and *NANOG*, were also verified in the same manner (Fig. 5d). Extracting the proteins from cell nucleases, YAP1 and stem-associated factors were upregulated in CBX4-overexpressed cells by western blot analyses (Fig. 5e). Therefore, the protein level of CBX4 and YAP1 were verified in the whole-cell lysis, cytoplastic lysis, and nuclear lysis. Although CBX4 did not result in the total protein of YAP1 increasing in SR whole cells, YAP1 could translocate from cytoplasm to nucleus according to the expression of CBX4 in SR cells (Fig. 6f). In addition, using flow cytometric analysis, the level of YAP1 expression in cytoplasm was decreased from about 35.8 to 1.4% in SR cells (Fig. 5g). And it can be rescued by knockdown of CBX4 in SR cells (Fig. 5h). It implicated that YAP1 expression in the nucleus increases with sorafenib resistance. Furthermore, expression of Flag-tagged CBX4 or Flag-tagged YAP1 in HEK 293FT cells showed that the reason of CBX4 addressing YAP1 or HIF1α to nuclear translocation was dependent on protein–protein interaction, as determined by immunoprecipitation and immunoblotting analyses (Fig. 5i). These data suggest CBX4 hereby affect the location of YAP1 protein but not the production.

### Targeting YAP and CBX4 inhibits tumour formation

To identify the functional role of CBX4 and YAP1 in HCC, the CBX4 inhibitor UNC3866 and the YAP1 inhibitor CA3 were used to evaluate tumour sphere formation in vitro. Both inhibitors reduced self-renewal ability of sorafenib-induced tumour formation in Huh7-SR cells (Fig. 6a). Moreover, associated proteins, such as HIF1α, YAP1, and stem-like genes associated with CSC properties (SOX2, NONAG, and BMI1), in the cell nucleus were tested by western blot (Fig. 6b). Furthermore, tumour growth in BALB/c nude mice was assessed after i.p. injection with 10 mg/kg/day UNC3866 and 1 mg/kg/day CA3. Both treatments significantly decreased Huh7-SR tumour growth (Fig. 6c). The combination of UNC3866 and CA3 dramatically inhibited sorafenib resistance based on tumour volume measurements collected every 2 days and the wet weight of the tumours (Fig. 6d, e). Double immunofluorescence staining for CBX4 and YAP1 expression in Huh7-SR tumours with or without treatment with their inhibitors was also conducted, and the expression of CBX4 and YAP1 was as prominent in the nucleus as it was in the cytoplasm in SR tumours. However, after treatment with the inhibitors, YAP1 expression was decreased in the nucleus after treatment with UNC3866, but YAP1 production in cytoplasm was not affected. Similarly, CA3 reduced the nuclear translocation of CBX4 but did not interfere with the CBX4 protein levels. When combined, UNC3866 and CA3 could dramatically downregulate CBX4 and YAP1 signalling (Fig. 6f). Additionally, the co-localisation of YAP1 and HIF1α in the nucleus in Huh7-SR cells was validated by immunofluorescence assay and the level of HIF1α was decreased by UNC3866 treatment (Fig. 6g). Altogether, these data confirm CBX4 and YAP1 as two targets of sorafenib resistance and indicated a key role in maintaining the CSC properties of HCC; this activity is illustrated in Fig. 6h.

## Discussion

Drug resistance is a serious cause of therapeutic failures in HCC. In addition to surgical treatment, sorafenib and regorafenib are two critical chemotherapies for patients whose disease progresses towards advanced HCC over a 10-year period or continuously for 3 years.^4,24^ Despite the proven efficacy of sorafenib to significantly increase OS in patients,^3^ the constantly effective responses for patients are not long appreciated for halting disease progression because advanced HCC often develops resistance to anti-proliferative therapies.^25^ In the present study, we established two drug-resistant cell lines to further explore the mechanism of sorafenib resistance with the goal of elucidating candidate targets for improving the efficacy of HCC therapy.

The relationship between CSC properties and sorafenib resistance has been predicted and highlighted in recent reports. CSC traits drive tumorigenicity in HCC cells and lead to HCC recurrence and sorafenib resistance.^26^ However, SR HCC is causally linked to the maintenance of stem-like properties.^27^ Enriched spheres with SR signatures have been related to CSCs, metastasis, and recurrence of HCC.^28^ These findings provide us with confidence in defining the correlation between SR cells and CSC traits as well as demonstrate the self-renewal and tumour formation of serially diluted transplanted SR HCC cells.

Recent studies have revealed that a series of miRNAs are involved in HCC tumour development. For example, miR367, miR223, miR494, miR221, and miR622 expression levels were increased with sorafenib resistance in HCC cells and associated with different pathways, such as the RAS-extracellular signal-regulated kinase (ERK) pathway, phosphoinositide-3 kinase (PI3K)-AKT pathway, mammalian target of rapamycin pathway, etc.^13–15,29,30^ However, miR-122 and miR137 expression significantly inhibited SR cells by reducing apoptosis through the AKT/ERK pathway^31^ and tumour-initiating cell phenotypes.^32^ Although these augmented miRNAs have been characterised to have anti-angiogenic, anti-metastatic, and anti-stem-like functions by targeting many transcription factors in HCC, we definitively identified miR424 as a tumour repressor that targets CBX4 and is associated with poor outcomes in HCC. A previous study revealed that miR424 significantly highlights the stem-cell-like properties of HCC, while the transcription factor PBX3 responds to this activity and modulates tumorigenesis.^17^ However, how miR424 is relevant to sorafenib resistance is unknown; thus, in this study, we further explored whether miR424 governs sorafenib resistance by directly targeting CBX4 and activating CBX4-induced tumour formation and self-renewal characteristics. As previously reported, sorafenib inhibited EGF receptor (EGFR) activity and directly affected the downstream PI3K-YAP pathway.^33^ Nevertheless, the interaction between PI3K and YAP has not been sufficiently explained. Based on the correlation between PI3K and miR424^34,35^ and this study, we give one explanation of PI3K/YAP interactivity through miR424–CBX4, which was induced by sorafenib resistance. But the mechanism of how miR424 was regulated by PI3K needs further understanding in the future.

Because we reported that cytoplasmic CBX4 protein levels indicated poor survival for HCC patients who undergo surgical resection,^10^ CBX4 was followed by analysis of a TCGA data set and GTEx bioinformatics with 11 public HCC expression data sets that covered approximately 3401 clinical samples (Fig. S3 and Table S8).^36^ We clearly confirmed that a high CBX4 level contributes not only to tumorigenesis but also to a more advanced stage of HCC. As a factor of poor prognosis, CBX4 increases the transcriptional activity of HIF1α and hypoxia-induced VEGF expression in HCC.^9^ In addition, CBX4 results in BMI1 recruitment via its E3 sumo ligase activity^37^ and has been shown to suspend proliferation and promote terminal differentiation.^8^ This differential activity might be influenced by individual characteristics of different organs, as it was found that CBX4 drives opposing behaviours in colorectal carcinoma metastasis.^38^ In our present study, as a tumour progenitor, CBX4 was shown to induce stem-cell-like properties and promote YAP1 nuclear translocation in a HIF1α-dependent manner. This outcome is based on the discovery from the KEGG analysis of the sequencing data between CBX4-overexpressing cells and control cells. Thus exciting results indicate several interesting pathways downstream of CBX4 signalling. For example, Wnt signalling has always enhanced CSC properties,^39^ Toll-like receptor signalling facilitates stem cell marker expression in HCC,^40^ and the PI3K-AKT pathway is a canonical signalling pathway downstream of EGFR to reduce tumour-initiating cell frequency.^41^ The Hippo-YAP pathway showed the greatest upregulation in response to CBX4 in our results. Although HIF1α and YAP1 have been widely reported and contribute to CSCs in HCC,^42,43^ we actually found that CBX4 regulates YAP1 signalling through YAP1 translocation rather than its production in SR HCC, and this mechanism implicates that the CBX4-mediated YAP1 nucleolus translocation is an important event for sorafenib resistance and even contributes to HCC therapy as shown in Fig. 6h.

The mounting evidence suggests that CSCs are particularly resistant to chemotherapy,^44^ and cells with sorafenib resistance maintain their CSC properties.^27^ Our previous findings indicated that YAP1 plays a critical role in CSC self-renewal and tumour formation and that suppressing YAP1 could be an effective way to prevent the maintenance of CSCs.^21^ In this study, we propose that targeting the CBX4-mediated YAP1 activity could be viable in treating CSCs and might be a novel strategy for SR HCC. Therefore, defining CBX4-YAP1 mediators of resistance to therapy is critical to better understand the relationship between CSC and sorafenib resistance.

We also investigated the effect of the combination of CBX4 and YAP1 inhibitors (UNC3866 and CA3) as a therapy for SR cells. Notably, the use of three targeted medicines for treating tumours is not available in the clinic. However, in our study, we provide effective candidates for patients who experience chemotherapy failure but can still benefit from a long-term treatment, even if they develop sorafenib resistance. In our opinion, we suggest three ways to maintain an available strategy for sorafenib resistance: transducing cells with miRNA424, inhibiting CBX4 expression, and arresting the Hippo-YAP pathway. We also propose a rational, biomarker-based clinical trial (using CBX4 and YAP1 overexpression to enrich the HCC patient cohort). We would also like to provide empirical therapeutic strategies for reducing sorafenib resistance by conducting in-depth molecular analyses of HCC. However, there are still several limitations in our work: the samples from parents who underwent sorafenib treatment in early stage of HCC were obtained with great difficulty; the patients who had the second hepatectomies were a few, epically after sorafenib therapy failing; the binding sites for protein interactions of CBX4 and YAP1 would be explored or predicted reliably; and the protein modification of YAP1 such as phosphorylation or ubiquitin would be considered.

In conclusion, our data demonstrated that CBX4 is often overexpressed in HCC, especially in response to sorafenib resistance. Its mRNA expression is controlled by miR424, which affects the proliferation capacity and tumour stem-like properties of the cells. Increasing miR424 induces CBX4 disaggregation and reduces the interaction of CBX4 and HIF1α, subsequently decreasing YAP1 nuclear translocation to downregulate oncogenesis and inhibit malignant characteristics. If CBX4 and YAP1 are both inhibited, the best anti-tumour effect is achieved in vitro and in vivo, particularly in SR cells. Thus these data provide compelling evidence that CBX4 serves as a novel anti-sorafenib resistance target and provide a strong rationale to explain the cause of CSC maintenance from SR cells. Importantly, these molecules are a promising therapeutic target for HCC and can be used to improve the outcome of patients.

## Supplementary information

supplementary information

## Data Availability

All original data are archived and stored at the PUCH, Beijing, China. The cell lines generated in this study and controls will be made available to other researchers upon request, including Huh7 and PLC sorafenib-resistant cell lines.
