# Supplementary material for: Inhibiting CBX4 efficiently protects hepatocellular carcinoma cells against sorafenib resistance
Source: Br J Cancer. 2021 Jan 21;124(7):1237–48. doi: 10.1038/s41416-020-01240-6 (PMC8007794; doi:10.1038/s41416-020-01240-6)
Supplement: Supplementary file 1 — supplementary information [file 41416_2020_1240_MOESM1_ESM.pdf]

Supplementary information:

Inhibiting CBX4 efficiently protects hepatocellular carcinoma cells against sorafenib resistance

WEI ZHAO[1§*], BO MA[2§], ZHIHUA TIAN[3], HAIBO HAN[4], JINTIAN Tang[5], BING DONG[3], GUO AN[6], BAOSHAN CAO[7*], BOQING WANG[5*]

Supplemental figure and table legends

Fig S1. Wound healing assay was performed between SR cells and parental cells, and the histograms show the migration of them with unpaired t test statistics; * *P*<0.05.

Fig S2. HE and immunohistochemistry of CBX4 expression in paraffin-embedded tumor tissues
a HE was performed on SR, SR-shCBX4 and SR-miR424 tumor slides. Bar =200 µm. b CBX4 was detected in different groups by IHC staining. Bar =100 µm.

Fig S3. CBX4 expression is closely associated with tumor malignancy, stage, and poor overall survival
a Transcriptome sequencing data analysis from the TCGA database and GTEx database summary by HHCCBD (http://www.lifeome.net/database/hccdb) showing the changes in CBX4 expression in primary tumor tissues (1968 samples) and adjacent normal tissues (1387 samples). b The correlation between CBX4 expression and tumor T stage in the TCGA samples. c The high CBX4 expression level contributes to the poor outcome of HCC patients based on the Kaplan-Meier curve analysis from The Human Protein Atlas Website 18.1 Version, cutoff: 16.38.

Table S1. PCR primer sequences

Table S2.
Information for antibodies used in Western blots (WB) & Immune-fluoresces (IF) & Immunohistochemistry (IHC)

Table S3: Tumorigenic cell frequency in each fraction of Huh7 and PLC cells with limiting dilution in NOD/SCID. *: *P*<0.05

Table S4: Comparative analysis of IC50 (µM) values of sorafenib between CBX4 and control, as well as CBX4 knockdown and control.

Table S5: the candidate miRNAs conserved with CBX4 from 3 published databank and sequencing data.

Table S6: Comparative analysis of IC50 (μM) values of sorafenib between miR424 and control, as well as miR424 TUD and scramble.

Table S7. Relationship between miR424 expression and pathological features in HCC patients

Table S8: mRNA level of CBX4 form analyzing bioinformatics integrative molecular database of hepatocellular carcinoma by 11 public TCGA and GTEx database. *: *P*<0.05.

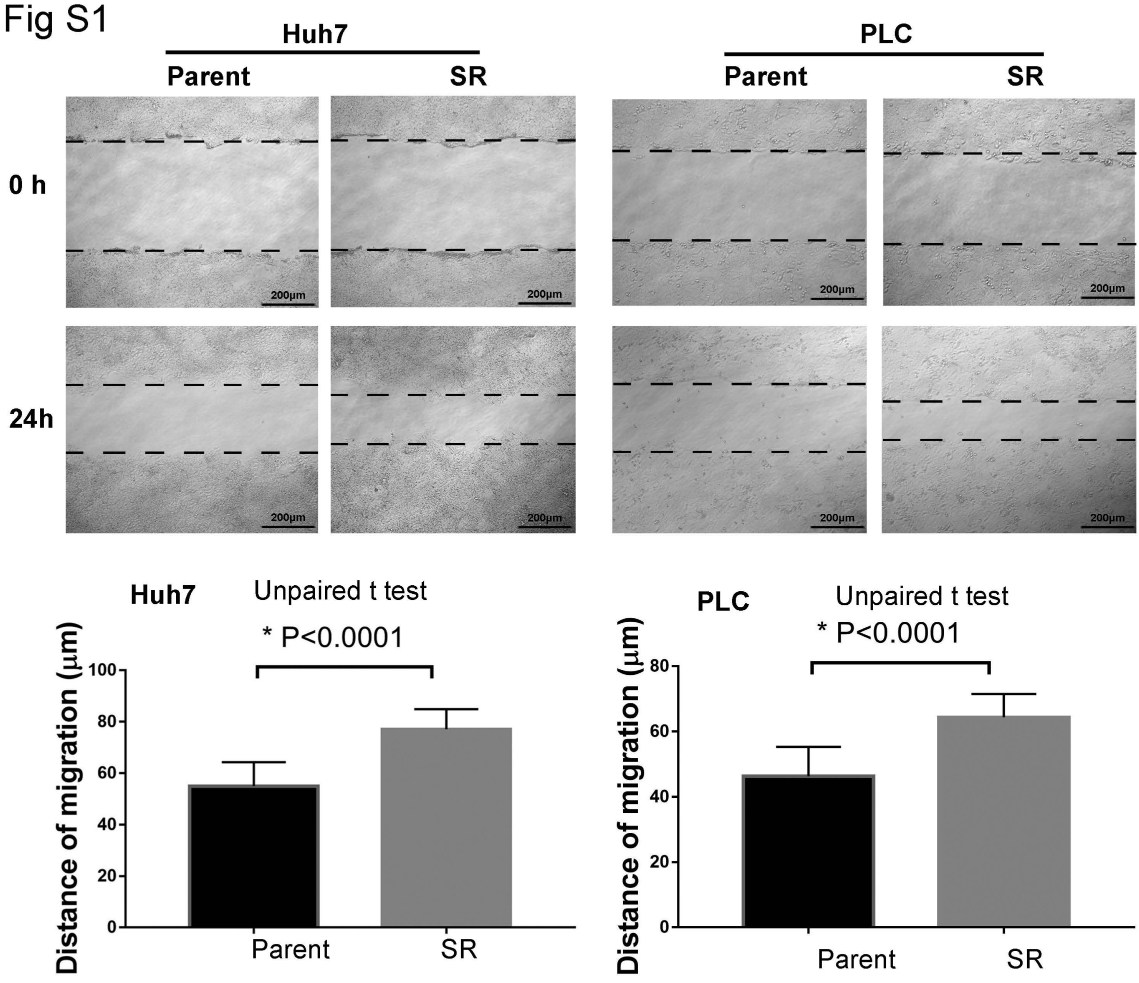
Fig S1
Huh7
PLC
Parent
SR
Parent
SR
0 h
24h
200μm
Huh7
Unpaired t test
* P<0.0001
Distance of migration (μm)
100
80
60
40
20
0
Parent
SR
PLC
Unpaired t test
* P<0.0001
Distance of migration (μm)
80
60
40
20
0
Parent
SR

Fig S2

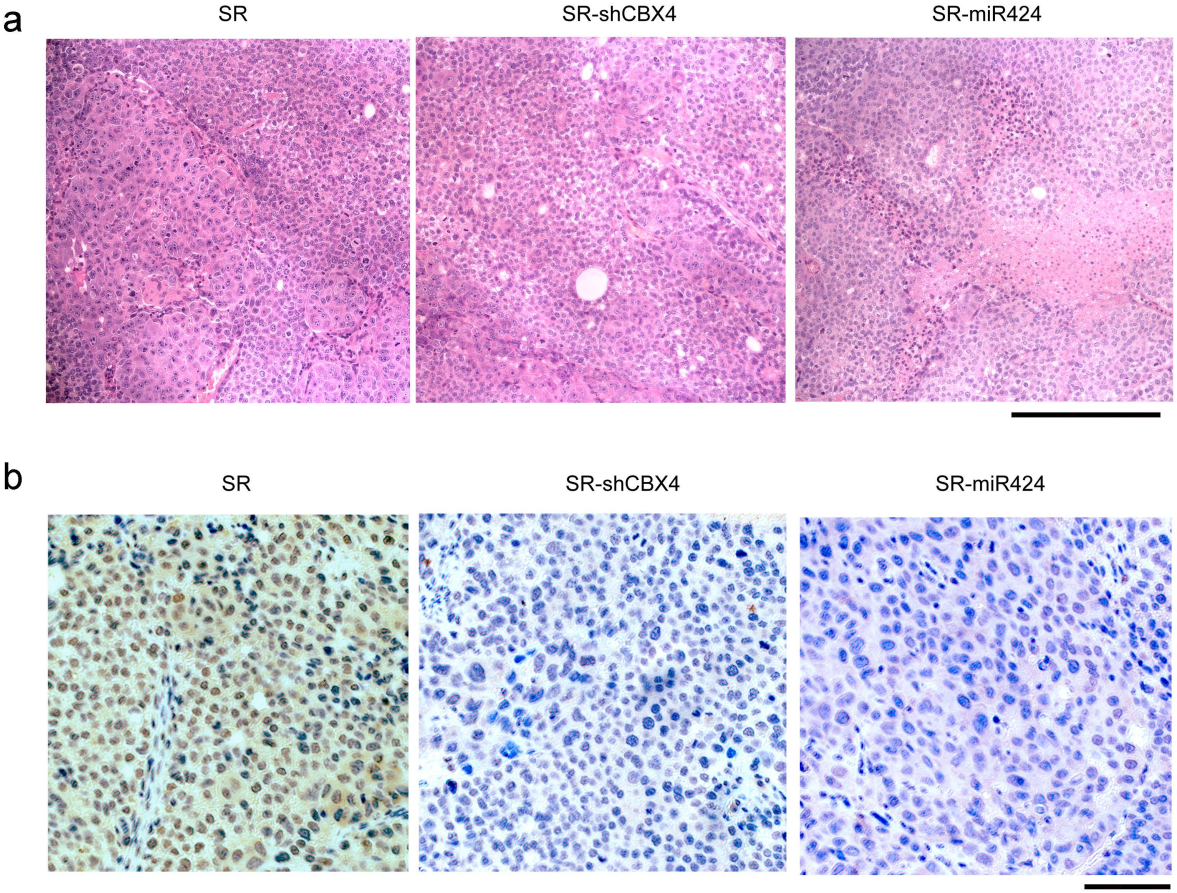

Fig S3

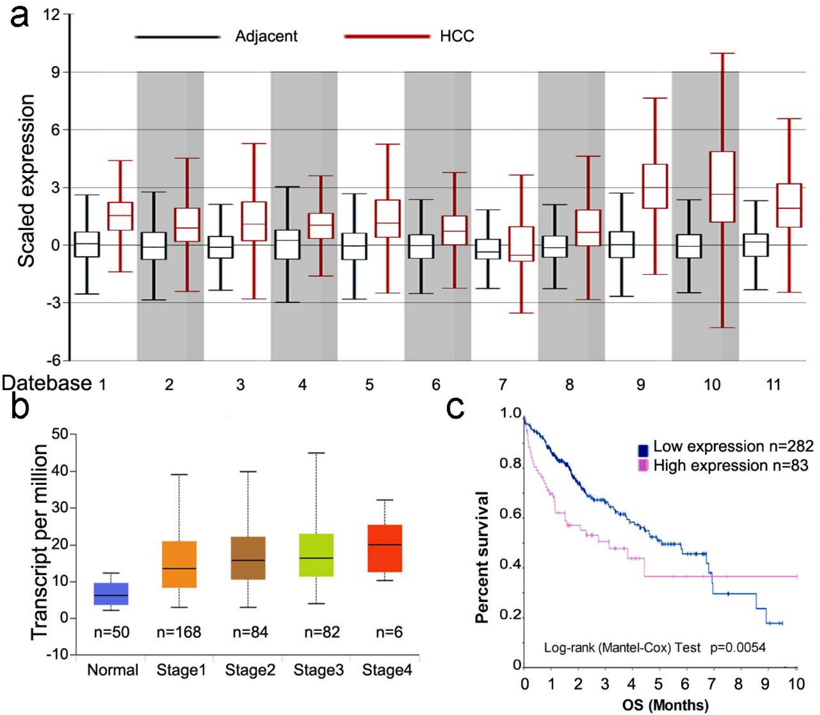
a
Adjacent
HCC
Scaled expression
12
9
6
3
0
-3
-6
Datebase 1 2 3 4 5 6 7 8 9 10 11
b
Transcript per million
50
40
30
20
10
0
-10
n=50
n=168
n=84
n=82
n=6
Normal
Stage1
Stage2
Stage3
Stage4
c
Low expression n=282
High expression n=83
Percent survival
1.0
0.8
0.6
0.4
0.2
0
Log-rank (Mantel-Cox) Test p=0.0054
0 1 2 3 4 5 6 7 8 9 10
OS (Months)

Table S1. PCR primer sequences

	qRT-PCR primers	
Gene	Sense	Anti-sense
ABCB1	5’-TGTCAAGGAAGCCAATGCCT-3'	5’-TCTGCCCACCACTCAACTGG-3'
ABCC1	5’-TGGGCAGGGATTCTCTTTTA-3'	5’-TCATGCTCACTTTCTGGCTG-3'
ABCG2	5'-GGAGGCCTTGGGATACTTTGAA-3'	5'-GAGCTATAGAGGCCTGGGGATTAC-3'
NANOG	5'-TGCCTCACACGGAGACTGTC-3'	5'-TGCTATTCTTCGGCCAGTTG-3'
OCT-4	5'-GACAACAATGAAAATCTTCAGGAGA-3'	5'-CTGGCGCCGGTTACAGAACCA-3'
SOX-2	5'-ACATGAACGGCTGGAGCAAC-3'	5'-AGGAAGAGGTAACCACAGGG-3'
CBX4	5'- GATATCCCATCAGCTGCTCC-3'	5'- GCTGGTCGCCCAAATATAAC-3'
YAP1	5'- CTGTCCCAGATGAACGTCAC-3'	5'- TTCTCTGGTTCATGGCAAAA-3'
NF-κB	5'- TTGCTGGTCCCACATAGTTG -3'	5'- ATGTATGTGAAGGCCCATCC -3'
PPARD	5'- CAGGGCTGACTGCAAACGA-3'	5'- CTGCCACAATGTCTCGATGTC-3'
GLI1	5'- GGCTCGCCATAGCTACTGAT -3'	5'- CCAGCGCCCAGACAGAG-3'
HES1	5'-TCAACACGACACCGGATAAAC-3'	5'-GCCGCGAGCTATCTTTCTTCA-3'
CTGF	5'-CAGCATGGACGTTCGTCTG-3'	5'-AACCACGGTTTGGTCCTTGG-3'
CYR61	5'-GGTCAAAGTTACCGGGCAGT-3'	5'-GGAGGCATCGAATCCCAGC-3'
ARRG	5'-GTGGTGCTGTCGCTCTTGATA-3'	5'-CCCCAGAAAATGGTTCACGCT-3'
BIRC5	5'-AGGACCACCGCATCTCTACAT-3'	5'-AAGTCTGGCTCGTTCTCAGTG-3'
GAPDH	5'-GACCCCTTCATTGACCTCAAC-3'	5'-CTTCTCCATGGTGGTGAAGA-3'

Table S2.

Information for antibodies used in Western blots (WB) & Immune-fluoresces (IF) & Immunohistochemistry (IHC)

Name	Vender	Cat No.	Species	Dilution
HIF1α	Abcam	ab51608	Rabbit monoclonal IgG	WB 1:2000<br>IF 1:200
CBX4	Santa Cruz	sc-517216	Mouse monoclonal IgG	IF 1:100
CBX4	BETHYL	A302-355A	Rabbit polyclonal IgG	IHC1:200
YAP1	Santa Cruz	sc15407	Rabbit polyclonal IgG	WB 1:200<br>IF 1:20
Nanog	Abcam	ab109250	Rabbit monoclonal IgG	WB 1:2000
SOX2	Abcam	ab97959	Rabbit polyclonal IgG	WB 1:2000
BMI1	Abcam	Ab126783	Rabbit monoclonal IgG	WB 1:5000
TAZ	Cell signaling	83669	Rabbit monoclonal IgG	WB 1:2000
Histone H3	Cell signaling	4499	Rabbit monoclonal IgG	WB 1:5000
GAPDH	Bioworld	BS606030	Rabbit polyclonal IgG	WB 1:20000

Table S3: Tumorigenic cell frequency in each fraction of Huh7 and PLC cells with limiting dilution in NOD/SCID. *: significant.

	Tumor Formation		Frequency	P value
	10^3	10^2	Tumorigenic cell (95%)	
Huh7-Control	3/5	2/5	1/711(1/1928-1/262)	*3.3E-5
Huh7-SR	5/5	5/5	1/1(1/125-1/1)	
PLC-Control	2/5	2/5	1/1061(1/3128-1/360)	*4.7E-6
PLC-SR	5/5	5/5	1/1(1/125-1/1)	

Table S4: Comparative analysis of IC50 (μM) values of sorafenib between CBX4 and control, as well as CBX4 knockdown and control.

	Huh7-C	Huh7-SR	PLC-C	PLC-SR
Control	1.52±0.18		2.5±0.39	
Cbx4	40.14±1.6*		32.7±0.5*	
Control		42.8±1.6		76.43±1.88
shCBX4		4.21±0.62*		2.75±0.44*

Each data point is represented as mean ± sd (n=4-6).

*P<0.01 Vs receptive Control

Table S5: The list of candidate upstream miRNAs of CBX4 and corresponding miRNAs sequencing data from sorafenib resistant cells.

Panel	Described
31 of predicted miRNAs conserved with CBX4 from 3 miRNA website (miRbase, TargetScan and miRanda)	miR-15; miR-16; miR-195; miR-424; miR-374; miR-410; miR-136; miR-495; miR-129; miR-181; miR-497; miR-144; miR-520; miR-200; miR-21; miR-3974; miR-371; miR-3177; miR-646; miR-429; miR-875; miR-4524; miR-6749; miR-216; miR-533; miR-101; miR-340; miR-8084; miR-366; miR-730; miR-518
44 of co-downregulated miRNAs both in Huh7-SR and PLC-SR cells	miR-8085; miR-1268; miR-6832; miR-26; miR-106; miR-615; miR-5582; miR-4519; miR-4285; miR-4667; miR-let-7; miR-1183; miR-619; miR-3657; miR-342; miR-365; miR-6784; miR-424; miR-320; miR-4999; miR-3127; miR-5703; miR-1289; miR-3909; miR-6826; miR-4456; miR-2666; miR-4660; miR-1204; miR-6851; miR-4632; miR-5585; miR-1304; miR-6129; miR-4743; miR-320; miR-4479; miR-4517; miR-4467; miR-4324; miR-574; miR-339; miR-4785; miR-93

miRbase: http://www.mirbase.org/;

TargetScan: http://www.targetscan.org/vert_71/ ;

miRanda: http://www.microrna.org/microrna/home.do

Table S6: Comparative analysis of IC50 (μM) values of sorafenib between miR424 and control, as well as miR424 TUD and scramble.

	Huh7-CBX4	Huh7-SR-shCBX4	PLC-CBX4	PLC-SR-shCBX4
U6	52.13±1.8		44.2±1.4	
MiR424	2.46±0.22*		3.21±0.53*	
Scramble		3.86±0.6		3.76±0.55
miR424 TUD		42.13±1.62*		54.3±1.56*

Each data point is represented as mean ± sd (n=4-6).
*P<0.01 Vs receptive Control(U6 and Scramble)

Table S7. Relationship between miR424 expression and pathological features in HCC patients

Variable	Case no.	miR424 expression[1] (RQ: $2^{-\Delta Ct}$) Median	Range	p^2	miR424expression[1] (ΔCt) Mean ± SEM	p^3
Gender						
Male	87	0.001	0.00001-3.24	0.79	9.32±4.06	0.71
Female	19	0.002	0.00002-1.21		9.51±3.95	
Age						
≤60	69	0.001	0.00001-2.11	0.74	9.15±5.26	0.78
>60	37	0.002	0.00001-3.24		9.71±5.88	
Size						
≤5	46	0.006	0.0015-0.95	0.0022*	9.34±2.1	0.0011*
>5	60	0.0022	0.0004-0.53		6.19±1.4	
Venous invasion						
Absent	41	0.0018	0.00001-0.08		8.58±2.19	
Present	56	0.00019	0.00002-0.04	0.81	8.95±2.12	0.42
No descripted	9	0.0011	0.00001-0.02		8.77±2.05	
Cirrhosis						
Absent	38	0.0915	0.00369-0.65	0.0001*	13.15±2.22	0.0001*
Present	68	0.0334	0.000433-0.95		6.16±1.65	

1 Quantified by qRT–PCR. miR424 was normalized to U6. 2 Mann–Whitney U test for the comparison between two groups or Kruskal–Walis test for more groups. 3 Unpaired Student’s t-test for the comparison between two groups or ANOVA for more groups.

Table S8: mRNA level of CBX4 form analyzing bioinformatics integrative molecular database of hepatocellular carcinoma by 11 public TCGA and GTEx database. *: Significate.

Dataset	P-value	Type	Numbers	Mean	STD
HCCDB1	*1.27E-22	HCC	100	9.808	0.5881
		Adjacent	97	9.001	0.3958
HCCDB2	*1.46E-49	HCC	268	1.834	0.7753
		Adjacent	243	0.947	0.27
		Cirrhotic	40	1.151	0.2792
		Healthy	6	1.075	0.2328
HCCDB3	*3.53E-68	HCC	240	7.776	0.5104
		Adjacent	193	6.953	0.2559
HCCDB4	*5.54E-14	HCC	225	4.171	0.4663
		Adjacent	220	3.876	0.3217
HCCDB5	6.73E-01	HCC	88	6.446	1.349
		Adjacent	48	6.357	1.067
HCCDB6	*7.39E-6	HCC	81	7.573	0.5912
		Adjacent	80	7.161	0.5347
HCCDB7	*5.22E-26	HCC	228	5.131	0.4459
		Adjacent	168	4.701	0.3076
HCCDB8	*8.9E-9	HCC	351	9.74	0.7207
		Adjacent	49	9.018	0.7128
HCCDB9	*2.05E-6	HCC	60	8.315	0.3905
		Adjacent	60	8.016	0.2405
HCCDB10	*9.37E-9	HCC	115	8.954	0.594
		Adjacent	52	8.461	0.4224
HCCDB11	*1.18E-37	HCC	212	3.577	0.6659
		Adjacent	177	2.652	0.6056
